# Novel Cationic Meso-Arylporphyrins and Their Antiviral Activity against HSV-1

**DOI:** 10.3390/ph14030242

**Published:** 2021-03-08

**Authors:** Kseniya A. Zhdanova, Inga O. Savelyeva, Artem V. Ezhov, Andrey P. Zhdanov, Konstantin Yu. Zhizhin, Andrey F. Mironov, Natal’ya A. Bragina, Alla A. Babayants, Irina S. Frolova, Nadezhda I. Filippova, Nadezhda N. Scliankina, Olga N. Scheglovitova

**Affiliations:** 1 MIREA—Russian Technological University, Vernadsky Prospect 86, Moscow 119571, Russia; inga.saveleva.96@mail.ru (I.O.S.); arx1mond@ya.ru (A.V.E.); zhizhin@igic.ras.ru (K.Y.Z.); mironov@mitht.ru (A.F.M.); n.bragina@mail.ru (N.A.B.); 2 Kurnakov Institute of General and Inorganic Chemistry of the Russian Academy of Sciences, Leninskii Pr. 31, Moscow 117907, Russia; zhdanov@igic.ras.ru; 3 Gamaleya Research Center of Epidemiology and Microbiology, Gamaleya Str. 18, Moscow 123098, Russia; babayants@gamaleya.org (A.A.B.); frolova@gamaleya.org (I.S.F.); gecnst105@gmail.com (N.I.F.); nvsklankina@yandex.ru (N.N.S.); scheglovitova@yandex.ru (O.N.S.)

**Keywords:** viruses, antiviral activity, synthesis, cationic porphyrins, anti-HSV-1, dark antiviral activity of porphyrins, Pluronic F127, nanovehicles

## Abstract

This work is devoted to the search for new antiherpes simplex virus type 1 (HSV-1) drugs among synthetic tetrapyrroles and to an investigation of their antiviral properties under nonphotodynamic conditions. In this study, novel amphiphilic 5,10,15,20-tetrakis(4-(3-pyridyl-*n*-propanoyl)oxyphenyl)porphyrin tetrabromide (**3a**), 5,10,15,20-tetrakis(4-(6-pyridyl-*n*-hexanoyl)oxyphenyl)porphyrin tetrabromide (**3b**) and known 5,10,15,20-tetrakis(1-methyl-4-pyridinio)porphyrin tetraiodide (**TMePyP**) were synthesized, and their dark antiviral activity in vitro against HSV-1 was studied. The influence of porphyrin’s nanosized delivery vehicles based on Pluronic F127 on anti-HSV-1 activity was estimated. All the received compounds **3a**, **3b** and **TMePyP** showed virucidal efficiency and had an effect on viral replication stages. The new compound **3b** showed the highest antiviral activity, close to 100%, with the lowest concentration, while the maximum **TMePyP** activity was observed with a high concentration; porphyrin **3a** was the least active. The inclusion of the synthesized compounds in Pluronic F-127 polymeric micelles had a noticeable effect on antiviral activity only at higher porphyrin concentrations. Action of the received compounds differs by influence on the early or later reproduction stages. While **3a** and **TMePyP** acted on all stages of the viral replication cycle, porphyrin **3b** inhibited viral replication during the early stages of infection. The resulting compounds are promising for the development of utilitarian antiviral agents and, possibly, medical antiviral drugs.

## 1. Introduction

Currently, viruses are becoming a global threat to all of humanity. Herpes viruses are widespread in the human population, and the infectious process they cause is characterized by a chronic latent course, with periods of virus reactivation and the manifestation of the disease [1,2,3]. A virus causes an acute disease of the mucous membranes of the oral cavity, cornea, gastrointestinal tract, urogenital tract and skin; in rare cases, it causes encephalitis and leads to severe neonatal infection [4,5]. Primary infection always leads to a lifelong virus persistence. A virus reaches the nerve ganglia from the primary focus along the nerve endings, and, in the neurons, the virus goes into latency-associated transcripts (LATs), which are accompanied by the development of latent infection, which can be reactivated [6]. Reactivation of the infection leads to relapse, and, in older people, the local inflammatory process, with viral latency in the neurons, can lead to the development of neurodegenerative diseases [7]. Herpetic infection also causes severe complications in immunocompromised patients, pregnant women and newborns [8,9]. According to the cryoelectron tomography, HSV-1 is an enveloped virus, with a sphere diameter of 186 nm and, including glycoprotein protrusions, a diameter of 225 nm [10]. Searches for antiherpetic agents to treat and prevent the diseases caused by HSV-1 have been carried out for many years [11]. However, the problem of finding new anti-HSV-1 agents and treatment methods continues to be relevant due to the development of resistance to existing drugs and the lack of a long-term effect from the use of specific prophylactic agents [12].

**Figure 1:**
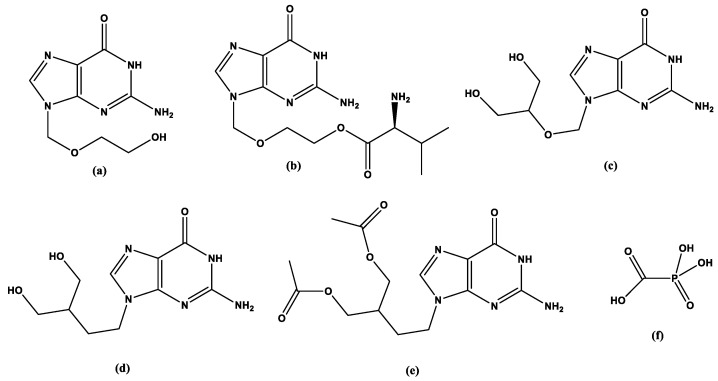
Antiherpetic drugs used in the clinic: (**a**) acyclovir, (**b**) valacyclovir, (**c**) ganciclovir, (**d**) penciclovir, (**e**) famciclovir, (**f**) foscarnet.

At present, modified nucleosides or their prodrugs are the most commonly used antiherpetic drug. The mechanism of their action is related to DNA polymerase suppression. The most famous drug is acyclovir (ACV) (Figure 1a), proposed in 1977 [13]. ACV is an effective and nontoxic drug, but it has low oral bioavailability and solubility, as well as a short lifetime in the bloodstream. A series of drugs with higher pharmacological properties has been received, for example, valacyclovir (L-valine ether of ACV, Figure 1b), ganciclovir (9-(1,3-dihydroxy-2-propoxymethyl)guanine, Figure 1c), etc. Non-nucleoside drugs include derivatives of 1,2,4-triazole[1.5-α]pyrimidine [14], hydroxyurea [15], foscarnet (Figure 1f), etc. It should also be noted that existing drugs do not relieve patients from the persistent nature of the disease, and the result of their long-term use may give rise to the emergence of resistant strains of the virus, which makes the course of the disease uncontrollable. In this regard, it is important to expand the list of possible antiherpetic drugs among biologically active compounds of various classes and to establish targets for their action.

Porphyrins are a unique class of heterocyclic tetrapyrrolic molecule. They play an essential role in vital biological processes of energy conversion and oxygen transfer. Interest in this class of compound is associated with a wide range of applications in various fields of medicine and technology, due to their unique photophysical properties (intense absorption in the visible and near-infrared regions, fluorescence properties, efficient generation of singlet oxygen, etc.) and their chemical and thermal stability [16,17,18,19,20,21,22,23,24]. Natural porphyrins and their synthetic analogues are widely used as photosensitizers for the photodynamic therapy (PDT) of neoplasms [25,26,27,28,29] and the photodynamic inactivation (PDI) of microorganisms, such as viruses [30,31,32,33,34], bacteria [35,36,37,38] and fungi [39,40].

Synthetic porphyrins with positive-charged groups have shown antiviral and antibacterial effects directly depending on their structure [31,33,35]. The cationic charge of the molecule promotes the electrostatic binding of porphyrin to the outer surface of the bacterial cell. DNA is also one of the most specific biological targets for cationic porphyrins. The formation of strong complexes of these compounds with nucleic acids has been reported, including for the recognition of noncanonical structures of DNA called G-quadruplexes [41]. Other authors [42] have shown that the HSV-1 genome contains multiple clusters of repeated G-quadruplex. In this regard, G-quadruplex binding ligands, such as porphyrins, could be envisaged as new therapeutic antiherpetic drugs. Porphyrins with pyridinium residues are especially interesting in this regard. The use of cationic 5,10,15,20-tetrakis (4-methylpyridine-4-yl)porphyrin (**TMePyP**) and its derivatives against HIV [33,43], bacteriophage MS2 [44], T7 [45] and hepatitis A virus [46] is also known. Elsewhere [31,32], the photoinduced activity of **TMePyP** derivatives against HSV-1 and HSV-2 have also been shown. The photodynamic virucidal activity of tetrapyrroles against HSV-1 is associated with phototoxic damage of the viral envelope proteins and unsaturated lipids and intracapsid components (nucleic acids and enzymes) [30]. However, a serious drawback of the photodynamic effect is its nonselectivity of cellular structures and the critically damaging action on cells due to reactive oxygen species (ROS). The literature describes few works dealing with the nonphotodynamic effect of tetrapyrroles on HSV-1 [30,31,32].

**Figure 2:**
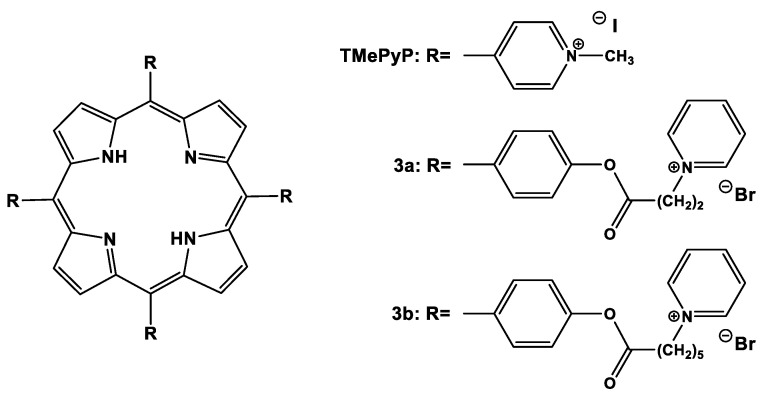
The structures of the investigated compounds.

In the current work, new amphiphilic cationic meso-arylporphyrins containing pyridinium residues at the periphery of the macrocycle were synthesized (Figure 2), and their antiviral effect against HSV-1 was investigated under nonphotodynamic conditions. The virucidal effect and the effect on HSV-1 Vero infected cells were evaluated. **TMePyP** was used as a reference compound, because of its widespread use in biological studies of tetrapyrrole compounds.

The hydrophobic nature of the tetrapyrrole macrocycle limits the use of these compounds in biomedicine. An effective approach to overcome this problem is the use of molecular delivery vehicles, such as liposomes, inorganic nanoparticles and polymers [22,25,27,47]. In recent years, researchers have shown increased interest in polymer micelles based on Pluronics—amphiphilic triple block copolymers of polyethylene oxide (PEO) and polypropylene oxide (PPO), several of which, including Pluronic F-127, have been approved for clinical use by the FDA [47]. Pluronics have found a broad application in PDT due to the ease of solution preparation, their nontoxicity, targeted delivery and the effective solubilization of hydrophobic tetrapyrroles. We have previously shown successful solubilization of tetraphenylporphyrin derivatives by Pluronic F-127 [48,49,50]. In this regard, another task of this work was to estimate the effect of Pluronic F-127 micelles on the porphyrins’ anti-HSV-1 activity.

## 2. Results and Discussions

### 2.1. Synthesis of Porphyrins

**Scheme 1:**
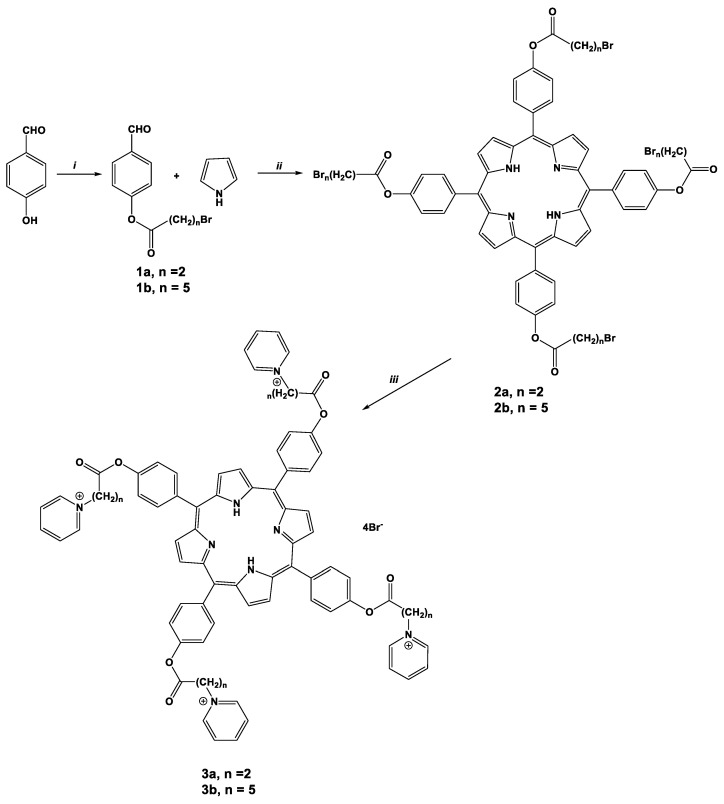
The general scheme of cationic porphyrin synthesis. Reagents and reaction conditions: (*i*) 3-Bromopropionyl chloride (**1a**) or 6-Bromohexanoyl chloride (**1b**), CHCl_3_, 0 °C; (*ii*) boron trifluoride etherate, chloroform, DDQ; yield 22% (**2a**) and 31% (**2b**); (*iii*) pyridine, reflux, 4h, yield 93–95%.

The literature mainly provides an investigation of cationic pyridinium porphyrins, analogues of the **TMePyP** [25,27,32,37,38,39,40]. In this work, the synthesized compounds differed in the length of aliphatic spacers between macrocycle and terminal cationic pyridinium groups (2 or 5 methylene units) (Figure 1, Scheme 1), in order to establish their structure/properties relationship. Porphyrins **2a,b** were synthesized using the method proposed by Lindsey [51], adapted in our laboratory for amphiphilic *meso*-arylporphyrins with alkyl substituents [52]. Pyrrole and substituted benzaldehydes **1a,b** were condensed with the formation of the bromine-substituted porphyrin precursors **2a,b** in 30–35% yields (Scheme 1). The target cationic porphyrins **3a,b** were obtained by boiling bromine-substituted porphyrins in pyridine to form a quaternary nitrogen atom in high yields (95–98%). The structure of the purchased products was confirmed by UV-vis, ^1^H and ^13^C NMR spectroscopy, elemental analysis and LCMS (ESI) mass spectrometry (Figures S3–S12, Supplementary Information).

### 2.2. Spectral Parameters and Photophysical Properties

The electronic absorption spectra (EAS) for the free-base compounds **3a,b** showed the Soret band at 415–418 nm and four Q-bands in the region at 519–656 nm. The normalized UV-vis spectra, as well as the fluorescence spectra in DMSO and ethanol, are shown in Figures S1 and S2 (Supplementary Information). Stokes shift values, quantum fluorescence yield and LogP coefficients are shown in Table 1. Fluorescence quantum yields were measured as compared with 5,10,15,20-tetrakisphenylporphyrin (TPP) (Φ*_F_* = 0.11) and ZnTPP (Φ*_F_* = 0.033). For free-base compounds **3a,b** fluorescence quantum yields were similar at ca. 0.13 in DMSO and ca. 0.14, respectively. Octanol-water distribution coefficients were calculated using 1-*n*-octanol and phosphate-buffered saline (PBS), according to the method described in [53]. For the compounds **3a,b**, negative Log*Pow* values were obtained, indicating that photosensitizers had a higher solubility in water than in the organic phase. As expected, compound **3a** was more hydrophilic (Log*Pow* = −4.86) than **3b** (Log*Pow* = −4.6). Such an effect can be associated with the increase in the number of methylene spacers in compound **3b**. The total data on photophysical parameters are presented in Table 1.

### 2.3. The Inclusion of Porphyrins in Pluronic F127 Micelles

**Figure 3:**
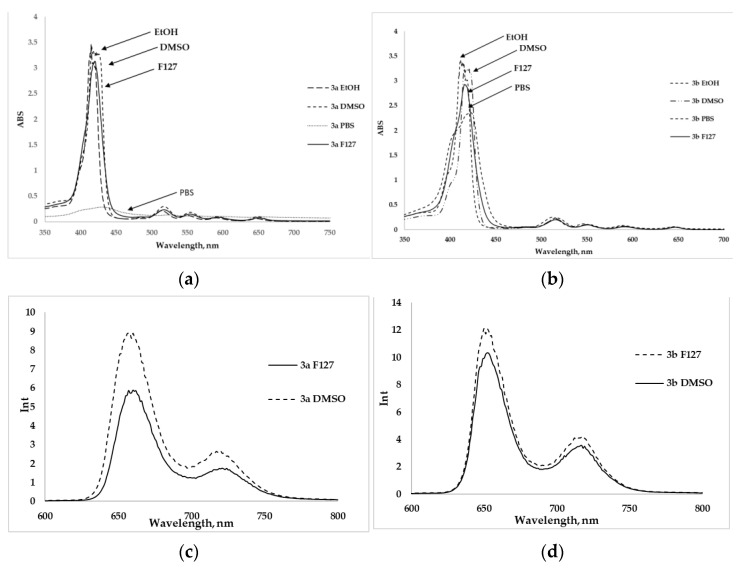
Electronic absorption spectra of compounds **3a** (**a**) and **3b** (**b**) (10^−6^ M) and fluorescence spectra in DMSO and Pluronic F-127 at the excitation λex = 415 nm for the compounds **3a** (**c**) and **3b** (**d**) (10^−6^ M).

Porphyrin macrocycles have demonstrated the tendency to aggregate in aqueous solutions due to the hydrophobic effect in combination with hydrogen and Van-der-Waals interactions [54,55,56,57]. Triblock copolymers, such as Pluronic, have been shown to reduce the self-aggregation of molecules and increase the solubility of porphyrins in aqueous media [58,59,60]. The obtained porphyrins **3a,b** have been incorporated into Pluronic F-127 polymer micelles using the thin-film method [60]. The obtained solutions of porphyrins **3a,b** in PBS have been studied spectrophotometrically to exclude aggregation traits, such as the broadening of absorption bands [48,49]. As can be seen from the UV-vis spectra compounds, **3a,b** tended to aggregate in a buffer solution (broadening and with a decrease in the intensity of the Soret band with a bathochromic shift of 10 nm for **3b** and 17 nm for **3a**), compared with solutions of these porphyrins in DMSO and ethanol. The monomeric form for porphyrins **3a,b** was observed when using Pluronic F-127 micellar formulation (Figure 3a,b). The fluorescence spectra of **3a,b** in DMSO and Pluronic F-127 showed a slight decrease in the intensity of the bands; however, fluorescence quenching was not observed, suggesting that porphyrins **3a,b** were in monomeric form (Figure 3c,d).

**Figure 4:**
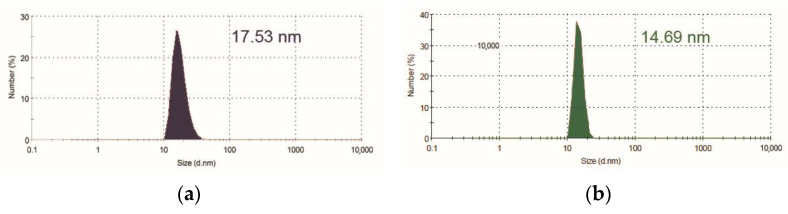
Particle sizes obtained by the dynamic light scattering (DLS) method for micellar solutions of compound **3a** (**a**) and **3b** (**b**) (concentration 3·10^−6^ M).

The size of obtained micelles for the compounds **3a,b** was determined by the dynamic light scattering (DLS) method. The micelle size for compounds **3a,b** was 17.53 nm and 14.69 nm, respectively, at concentrations of 3·10^−6^ M (Figure 4a,b). It has previously been reported that a similar micelle size increased the efficiency and circulation of the encapsulated drug in the blood due to the small scale, which avoided the capture of nanoparticles by the reticular endothelial system [61].

### 2.4. Virucidal Activity

The ability of porphyrins to directly inactivate microorganisms may depend on their degree of solubility [50]. Therefore, in this work, the anti-HSV-1 activity of compounds **3a,b** and **TMePyP** was compared in PBS (pH = 7) and Pluronic F-127 micelles. 

Before testing antiviral activity, the effect of porphyrins dissolved in PBS and Pluronic F-127 on the viability of *Vero* cells was evaluated. Cells were incubated for 72 h using different concentrations of porphyrins (0.39–100 μM). Viability was assessed using the MTT test (Figure S13, Table S1, Supplementary Information). The viability of *Vero* cells depends on the porphyrins’ structure and formulation. The Pluronic F-127 and PBS formulations of compounds **3a** and **TMePyP** showed low toxic effects: the maximum used concentrations of 100 μM prevented the determination of CC_50_ (Table 2). Micellar formulation of **3b** led to its higher toxicity, compared to the phosphate buffer, and CC_50_ values were 27.0 µM and 43.0 µM, respectively.

**Figure 5:**
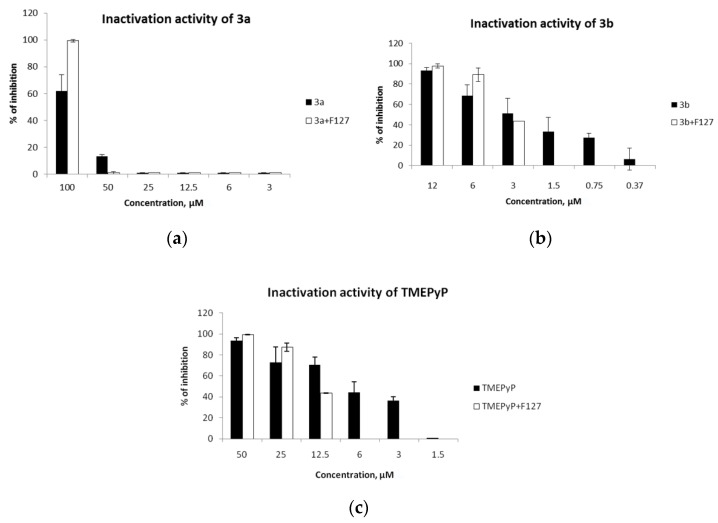
Virucidal activity of **3a** (**a**), **3b** (**b**), **TMePyP** (**c**) dissolved in PBS and Pluronic F-127 regarding HSV-1. The results are presented as the virus titer percentage, with the porphyrin regarding the virus titer in control, which was taken as 100%. Each point represents the mean ± deviation of three experiments with three replicates.

Next, the virucidal effect of the drugs on HSV-1 in PBS and Pluronic F-127 was studied, in accordance with CC_50_ (Figure 5). The virucidal activity tests were performed by incubating HSV-1 with different concentrations of **3a,b** and **TMePyP** for 2 h at a temperature of 36.8 °C and 5% CO_2_. Untreated viral suspensions were used as a control. Then, samples were added to a *Vero* culture seeded in 96-well plates, and the residual virus content was evaluated by developing a cytopathic effect on the fourth day of cultivation.

The toxic concentration for compound **3a** was >100 μM. Porphyrin **3a** showed a virucidal effect only at the maximum concentration of 100 μM, while the inhibition of the virus was more pronounced when using Pluronic F-127 micelles than with the use of PBS.

Porphyrin **3b** exerted a virucidal effect starting at 12 μM. Antiviral activity of this compound depended on the solubilizing form used. Thus, dissolution of **3b** in PBS revealed virucidal activity in concentrations from 12 μM to 0.75 μM; its activity using Pluronic F-127 was at concentrations from 12 μM to 3 μM.

The toxic concentration of **TMePyP** was >50 μM; therefore, a concentration of 50 μM and lower values were used to detect its virucidal effect. The virucidal effect of **TMePyP** in PBS was manifested in a wide range of concentrations, from 50 μM to 3 μM; when using a **TMePyP** micellar solution, this effect was revealed in a narrower range of concentrations, from 50 μM to 12.5 μM. For all studied compounds, dissolution in Pluronic F-127 exerted a virucidal effect on HSV-1 only at high concentrations, while, in PBS medium, porphyrins were active in a broader range of concentrations. Perhaps this is because porphyrins encapsulated in Pluronic micelles at low concentrations cannot contact virions.

### 2.5. Antiviral Activity

**Figure 6:**
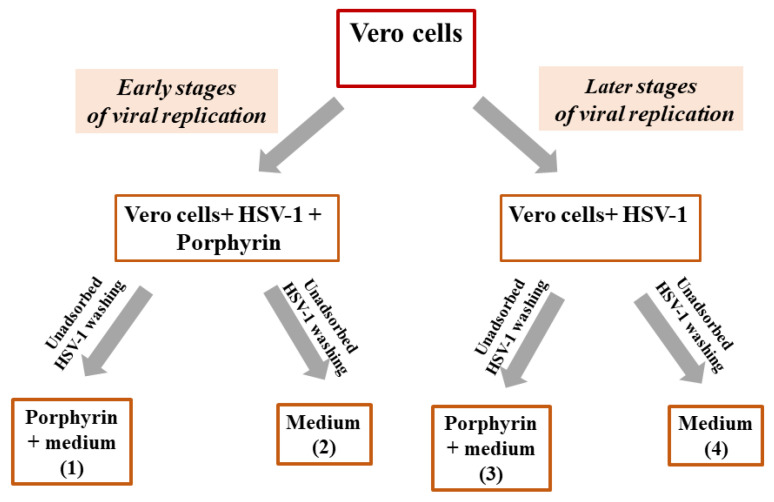
Experiment by the test compounds evaluation on the early or late stages of HSV-1 replication.

Porphyrin’s antiviral action was then determined in terms of their effect on the HSV-1 replication stages in Vero cells. A two-part experiment was conducted to evaluate the influence of the obtained compounds on the early or late stages of replication (Figure 6). First, the effect of porphyrins on the initial stages of reproduction was estimated. Thus, HSV-1 at MOI 0.1 was adsorbed on Vero cells in a medium containing the studied porphyrin; then the cell monolayers were washed from the nonadsorbed virus, and porphyrins of the same concentration were introduced into the plate (1) (Vero Cells + HSV-1 + Porphyrin, left arrow. Figure 6), or the cell monolayers were washed from the nonadsorbed virus, and the medium without the test compound was introduced into the cell monolayers microwell plate (2) (Porphyrin + HSV-1 + Porphyrin medium, right arrow. Figure 6).

**Figure 7:**
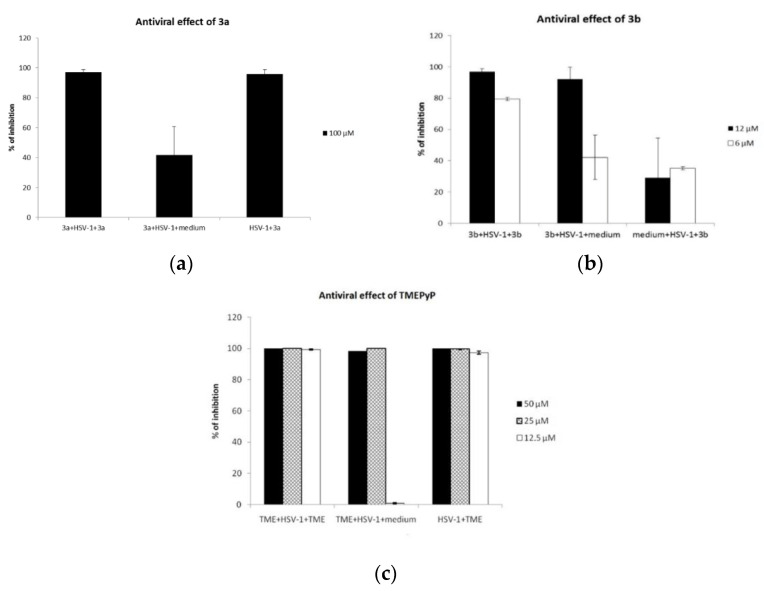
Inhibitory effect of porphyrins on the HSV-1 replication. The Vero cells were treated with compounds **3a** (100 µM) (**a**), **3b** (12 µM and 6 µM) (**b**) and **TMePyP** (50 µM, 25 µM and 12.5 µM) (**c**) simultaneously with HSV-1 (MI 0.1) or after HSV-1 (MI 0.1) adsorption. The results are presented as the percentage of the virus titer with porphyrin relative to its titer in the control (without porphyrin), which was taken as 100%. Each bar represents the mean ± deviation. Three experiments were performed with four repetitions.

The influence of porphyrin antiviral activity on the HSV-1 postadsorption stages was simultaneously evaluated in this experiment. The studied compounds were introduced after HSV-1 adsorption on Vero cells and washing of nonadsorbed virus particles (3) (Vero Cells +HSV-1, left arrow. Figure 6) or, after virus adsorption and nonadsorbed virus particles washing, only the medium was introduced (control of virus reproduction) (4) (Vero Cells +HSV-1, right arrow. Figure 6). After the formation of a pronounced cytopathic effect of the virus in control cultures (48 h of cultivation), the virus infection activity of all the samples was determined by titration in a Vero cell culture (Figure 7).

Compound **3a** suppressed the reproduction of the virus at the simultaneous introduction of the virus and the washing of the nonadsorbed virus (**3a** + HSV-1 + **3a**) or in the post-infection study (HSV-1 + **3a**) (Figure 7). Apparently, **3a** acts on both the initial and postadsorption stages of virus replication. The introduction of **3a** into the medium only at the cell infection stage (**3a** + HSV-1 + media) did not significantly suppress the reproduction of the virus.

The **TMePyP** compound also affected both the initial and late stages of virus replication. In addition, for this compound, a dose-dependent effect was noted: the simultaneous addition of **TMePyP** at high concentrations (50 μM and 25 μM) with the virus completely suppressed its replication (Figure 7). Conversely, a lower concentration of **TMePyP** (12 μM) did not suppress the HSV-1 reproduction when the porphyrin was introduced at the stage of virus adsorption.

Porphyrin **3b** had a greater effect on the early stages of virus reproduction (**3b** + HSV-1 + **3b**, **3b** + HSV-1 + medium), since suppression of reproduction was more pronounced when treating cells with porphyrin at the stage of virus adsorption on Vero cells (Figure 7). The effect was less pronounced at low concentrations of **3b**. Summarizing the experimental results, we can conclude that the final effect of the substances on HSV-1 was associated with both virucidal and antiviral effects.

## 3. Materials and Methods

### 3.1. Cells

African green monkey kidney cells (Vero) were cultured in DMEM (Dulbecco’s Modified Eagle Medium) supplemented with 5% fetal calf serum (FCS) and 50 µg/mL gentamycin, both purchased from Gibco (Thermo Fisher Scientific, Inc., Waltham, MA, USA), and maintained at 36.8 °C in 5% CO_2_ atmosphere.

### 3.2. Virus

HSV-1 (strain RD from WHO laboratory, Geneva, Switzerland) was propagated in Vero cells after a multiplicity of infection (MOI) of 0.1 TCID_50_/cell in DMEM-1% fetal calf serum (FCS) for 20 h. Then the supernatant was centrifuged at 700× *g* to remove cellular debris and stored in aliquots at −30 °C. To determine viral activity, tenfold dilutions of the virus were applied to the Vero cell monolayers in 96-well plates, and, after 4 days of cultivation at 36.8 °C in 5% CO_2_ atmosphere, titers were calculated based on the development of CPE (Cytopathic effect).

### 3.3. Cell Viability 

Cell viability was assessed by MTT reduction assay. Briefly, confluent monolayer Vero cells in 96-well plates (15000 cells/well) were incubated for 72 h with different concentrations of the compounds ranging from 0.39 µM to 100 µM in DMEM-1% FCS at 36.8 °C in 5% CO_2_ atmosphere. Next, cells were incubated for 1 h with 1.0 mg/mL of 3-(4,5-dimethylthiazol-2-y1)-2,5-diphenyltetrazolium bromide (MTT, PanEco, Moscow, Russia) at 36.8 °C, and then the solution was removed, and precipitated formazan was diluted in DMSO (PanEco, Moscow, Russia). The solution was read on a spectrophotometer at 540 nm. For each concentration, the average cell viability was calculated from the data of three experiments with three replicates each and was expressed as a percentage compared to the untreated cells (100%).

### 3.4. Virucidal Effect

HSV-1 at tenfold dilutions DMEM-1% FCS (900 µL) was incubated with different concentration of the compounds (100 µL) at the final concentration ranging from 3 µM to 100 µM in DMEM-1% FCS or without of compound, each sample in volume 1 mL, for 2 h at 36.8 °C in 5% CO_2_ atmosphere. After treatment, the samples were applied to the Vero cell (the day before, cells were seeded at the rate of 15,000 cells/well in 100 of DMEM-5% FCS in 96-well plates) monolayers in 96-well plates (100 µL/well), and, after 4 days of cultivation at 36.8 °C in 5% CO_2_ atmosphere, titers were calculated based on the development of CPE, and the inhibitory activity of each compounds was calculated in relation to the control (HSV-1 without compounds). Data are reported as means of three independent assays, each run in triplicate.

### 3.5. Influence of the Compounds on Virus Yield during Infection

Four variants were used to test compound efficiency in controlling development of infection in Vero cells. The Vero cells were seeded in 24-well plate in 1 mL of DMEM-5% FCS. (1) The cell monolayers were treated simultaneously with HSV-1 (MOI 0.1) in DMEM-1% FCS (900 µL) and investigated compounds in DMEM-1% FCS (100 µL) for 2 h at 36.8 °C in 5% CO_2_ atmosphere; then the cell monolayers were washed from the nonadsorbed virus and treated with 900 µL of DMEM-1%FCS and the same concentrations of compounds (100 µL). (2) The cell monolayers were treated simultaneously with HSV-1 (MOI 0.1) DMEM-1% FCS (900 µL) and compounds DMEM-1%FCS (100 µL) for 2 h at 36.8 °C in 5% CO_2_ atmosphere, and then the cell monolayers were washed from the nonadsorbed virus, after which only DMEM-1% FCS (1 mL) was added. (3) The cell monolayers were treated with HSV-1 (MOI 0.1) DMEM-1% FCS (900 µL) and DMEM-1% FCS (100 µL) for 2 h at 36.8 °C in 5% CO_2_ atmosphere and washed from the nonadsorbed virus; then DMEM-1% FCS (900 µL) and the same compounds in DMEM-1% FCS (100 µL) were added. (4) Control: the cell monolayers were treated with HSV-1 (MOI 0.1) in DMEM-1% FCS (900 µL), and DMEM-1% FCS (100 µL) was added for 2 h at 36.8 °C in 5% CO_2_ atmosphere and washed from the nonadsorbed virus, and then DMEM-1% FCS (1 mL) was added. In all variants, the treated cells were then incubated at 36.8 °C in 5% CO_2_ atmosphere.

After the development of a cytopathic effect in control cultures (HSV-1 + medium, 48 h), the virus content was determined in samples of the culture medium by applying ten-fold dilutions (100 µL) on a monolayers of Vero cells in wells of 96-well plates. After three days, virus titers were counted, and the percentage of inhibition regarding the titer of the virus in the control, which was taken as 100%, was determined. The results were presented as the average of three experiments of four replays.

### 3.6. Synthesis

#### 3.6.1. Reagents

All reagents were commercially available and used as received if not specially mentioned. All solvents were dried, as recommended in standard procedures. Chromatographic purifications were carried out on silica gel (Silica 60 0.04–0.063 mm/230–400 mesh, Macherey–Nagel, Dueren, Germany). Thin-layer chromatography was performed using aluminum sheets coated with silica gel 60 F254 (Macherey–Nagel, Dueren, Germany). TMеPyP was received according to the known procedure [62,63]. 

#### 3.6.2. Measurements

NMR (^1^H and ^13^C) spectra of the test solutions in CDCl_3_ or (CD_3_OD) were recorded on a Bruker MSL-300 pulse Fourier transform spectrometer (Bruker, Billerica, MA, USA) at frequencies of 300.3 and 75.49 MHz, respectively, with internal deuterium stabilization. Tetramethylsilane was used as an external standard. IR spectra (4000–500 cm^–1^) were recorded on Infralyum FT 02 Fourier spectrometer (Lumex Instruments Research and Production Company, Moscow, Russia) with a resolution of 1 cm^−1^. Samples were prepared as KBr tablets. Dynamic light scattering was measured using Malvern Zetasizer Nano ZS (Malvern, United Kingdom). Elemental analysis was performed on a C, H, N, S FLASH EA 112 analyzer of Termo Finnigan (Monza, Italy). Mass spectra were recorded using an 1100 LCMSD liquid chromatograph (Agilent Technologies, Santa Clara, CA, USA) equipped with a mass spectrometric chemical ionization detector at atmospheric pressure (HIAD, APCI, ESI) and with a UV spectrophotometric detector (DAD). Chromatographic separation was carried out on a Peekescientific 50 × 4.6 mm Hi-QC18 column (Glasgow, United Kingdom) with silica gel modified with an octadecyl phase (C_18_) with a sorbent particle size of 5 μm and a pore size of 50 Å (eluent A: 2.5% acetonitrile, 0.1% trifluoroacetic acid/water; eluent B: 0.1% trifluoroacetic acid/acetonitrile; from 10% eluent B linear gradient increase in 2.9 min to 100% B, then washing the column with 100% eluent B for 1 min, then balancing to the initial conditions for 0.1 min; mobile phase velocity 4 mL/min). Electronic absorption spectra were recorded using Shimadzu UV-1800 (Kioto, Japan) spectrophotometer at room temperature in quartz cells with an optical path length of 10 mm. Steady-state fluorescence measurements were carried out using Cary Eclipse (Agilent Technologies, Santa Clara, CA, USA) luminescence spectrometer at room temperature in 10 mm quartz cells with the excitation wavelength corresponding to the absorption maximum of the porphyrin in the Soret band region (415 nm). 

#### 3.6.3. General Procedure for the Substituted Benzaldehydes **1a,b** Preparation

To a solution of *p*-hydroxybenzaldehyde 1.2 g (9.83 mmol) and DMAP 0.9 g (7.37 mmol) in dichloromethane (25 mL), a solution of 2.00 g (11.8 mmol) 3-bromopropionyl chloride (a) or 2.52 g (11.8 mmol) 6-bromohexanoyl chloride (b) in 10 mL of dichloromethane (20 mL) were added dropwise during 30 min at 0 °C. After 30 min, the mixture was additionally stirred at room temperature for 24 h. Excess of solvent was removed under reduced pressure. The reaction mass was purified by column chromatography on silica gel G 60 (dichloromethane: hexane = 4:1). 

**4-(3-Bromo-*n*-propanoyloxy)benzaldehyde (1a).** Yield: 3.58 g (70%). Rf 0.6 (CH_2_Cl_2_); IR (ν, cm^−1^): 2928, 2849 (CH_2_)_2_, 1675 (C=O), 1467 (Ar), 1160, 1138 (C–O), 642, 550 (C–Br). ^1^H NMR (CDCl_3_, δ, ppm): 2.97 (2H, t, *J* = 6.65 Hz, CH_2_Br), 3.55 (2H, t, *J* = 6.65 Hz, OCOCH_2_) 7.46 (2H, d, *J* = 8.40 Hz, 3,5-(ArH)), 7.96 (2H, d, *J* = 8.70 Hz, 2,6-(ArH)), 9.99 (1H, s, CHO).

**4-(6-Bromo-*n*-hexanoyloxy)benzaldehyde (1b).** Yield 2.35 g (80%), Rf 0.3, (dichloromethane: hexane = 7:3); IR (ν, cm^−1^): 2926, 2848 (CH_2_)_5_, 1676 (C=O), 1467 (Ar), 1160, 1138 (C–O), 642, 550 (C–Br). ^1^H NMR: (CDCl_3_, δ, ppm): 1.55–1.65 (2H, m, OCO(CH_2_)_2_CH_2_(CH_2_)_2_Br), 1.77–1.84 (2H, m, OCOCH_2_CH_2_), 1.89–1.97 (2H, m, CH_2_CH_2_Br), 2.64 (2H, t, *J* = 7.24 Hz OCOCH_2_), 3.46 (2H, t, *J* = 6.69 Hz, CH_2_CH_2_Br) 7.51 (2H, d, 2,6-(ArH)), 7.96 (2H, d, 3,5-(ArH)), 10.01 (1H, s, CHO).

#### 3.6.4. General Procedure for the Porphyrins **2a,b** Preparation

Pyrrole 0.1 g (1.42 mmol) and 4-(3-bromo-*n*-propanoyloxy)benzaldehyde 0.38 g (1.50 mmol) 1a or 4-(6-bromo-*n*-hexanoyloxy)benzaldehyde 0.45 g (1.50 mmol) **1b** were dissolved in chloroform (100 mL). The reaction mixture was saturated with argon and stirred at room temperature for 5 min, after which 20 μL (0.15 mmol) of boron trifluoride etherate and 200 μL of absolute ethanol were added. The reaction mass was stirred under inert atmosphere at room temperature for 2 h. After that, argon was removed and 0.3 g DDQ (1.35 mmol) was added, and the resulting mixture was stirred at room temperature for 4 h. The reaction mixture was concentrated under reduced pressure. Oligomeric products were separated by flash chromatography on silica gel G 60 eluted with chloroform. The target product was purified by column chromatography on silica gel G 60 eluted with a system of chloroform:hexane = 4:1.

**5,10,15,20-tetrakis(4-(3-bromo-*n*-propanoyl)oxyphenyl)porphyrin (2a).** Yield: 0.09 g (22%). Rf 0.5 (CH_2_Cl_2_). EAS, λmax, nm (ε × 10^−3^): 417 (318.14); 515.5 (14.89); 550.8 (6.15); 590.1 (3.49); 646.5 (3.01). ^1^H NMR (CDCl_3_, δ, ppm): −2.74 (2H, c, NH-pyrrole), 3.38 (8H, t, *J* = 6.65 Hz, OCOCH_2_CH_2_Br), 3.86 (8H, t, *J* = 6.69 Hz, OCOCH_2_CH_2_Br), 7.57 (8H, d, *J* = 8.34 Hz, 3,5-(ArH)), 8.30 (8H, d, *J* = 8.30 Hz, 2,6-(ArH)), 8.95 (8H, s, CH-pyrrole). Elemental analysis: found, %: C 56.18; H 3.25; N 4.75. C_56_H_42_N_4_O_8_Br_4_; calculated, %: C 55.20; H 3.47; N 4.60.

**5,10,15,20-tetrakis(4-(6-bromo-*n*-hexanoyl)oxyphenyl)porphyrin (2b).** Yield 0.15 g (31%); Rf 0.8 (CHCl3). EAS, λmax, nm (ε × 10^−3^): 419.8 (321); 514.4 (15.28); 549.8 (5.96); 589 (3.63); 645.6 (3.43). ^1^H NMR (CDCl_3_, δ, ppm.): 1.56 (8H, m, CH_2_CH_2_Br), 1.98–2.05 (16H, m, OCOCH_2_(CH_2_)_2_), 2.79 (8H, t, *J* = 7.34 Hz, OCOCH_2_), 3.52 (8H, t, *J* = 6.69 Hz, CH_2_CH_2_Br), 7.54 (8H, d, *J* = 8.44 Hz, 3.5-(ArH)), 8.20 (8H, d, *J* = 8.48, Hz 2.6-(ArH), 8.88 (8H, s, CH pyrrole). ^13^C NMR (CDCl_3_ δ, ppm): 171.7, 145.5, 145.5, 138.6, 134.5, 127.6, 119.5, 61.0, 33.3, 30.8, 25.0, 23.6. Elemental analysis: found, %: C 58.18; H 4.65; N 4.10. C_68_H_66_N_4_O_8_Br_4_; calculated, %: C 58.89; H 4.80; N 4.04. 

#### 3.6.5. General Procedure for the Preparation of Porphyrins **3a,b**

The initial porphyrin **2a** (0.02 g, 0.016 mmol) or **2b** (0.05 g, 0.036 mmol) was dissolved in dry pyridine and boiled for 3 h. The formed precipitate was filtered and washed with chloroform; the solvent was removed under reduced pressure. The reaction product was isolated by recrystallization from diethyl ether. Yield 95–97%.

**5,10,15,20-tetrakis(4-(3-pyridyl-*n*-propanoyl)oxyphenyl)porphyrin tetrabromide (3a).** Yield: 0.019 g (95%). Rf 0.2 (CH_2_Cl_2_:EtOAc = 1:1); EAS, λmax, nm (ε × 10^−3^): 415.5 (312.5); 512.5 (14.5); 547.5 (6.2); 589 (3.7); 645.5 (3.5). ^1^H NMR (CD_3_OD, δ, ppm.): 3.68 (8H, t, *J* = 6.16 Hz, OCOCH_2_), 5.15 (8H, t, *J* = 6 Hz, CH_2_CH_2_Py), 7.35 (8H, d, *J* = 8.13 Hz, 3,5-(ArH)), 7.94 (8H, d, *J* = 8 Hz, 2,6-(ArH)), 8.19 (8H, t, *J* = 7.72 Hz, 3,5-Py), 8.59–8.69 (12H, m, 4-Py^+^CH pyrrole), 9.25 (8H, d, *J* = 5.66 Hz, 2,6-Py). ^13^C NMR (CD_3_OD δ, ppm): 171.4, 150.7, 145.8, 144.7, 139.4, 135.0, 128.3, 119.6, 119.2, 60.7, 30.2, 26.1. Elemental analysis: found, %: C 60.98; H 4.13; N 7.09, C_76_H_62_N_8_O_8_Br_4_ calculated, %: C 59.47; H 4.07; N 7.30. LCMS (ESI) *m*/*z*: 302.1 [M^+^4Br^−^]^+^; the molecular formula of C_76_H_62_N_8_O_8_ requires 303.6.

**5,10,15,20-tetrakis(4-(6-pyridyl-*n*-hexanoyl)oxyphenyl)porphyrin tetrabromide (3b).** Yield 46 mg (93%). EAS, λmax, HM (ε × 10^−3^), (CH_3_OH): 415 (328); 513 (13.37), 546 (6.07), 589 (3.64), 645 (3.56). ^1^H NMR (CD_3_OD, δ, ppm.): 1.72 (8H, m, OCO(CH_2_)_2_CH_2_(CH_2_)_2_Py), 2.01 (8H, m, OCOCH_2_CH_2_), 3.35 (8H, m, CH_2_CH_2_Py), 3.99 (8H, br s, OCOCH_2_), 4.36 (8H, m, CH_2_CH_2_Py), 7.04 (8H, m, 3,5-(ArH)), 7.83 (8H, d, 2,6-(ArH)), 7.96 (8H, m, 3,5-Py), 8.40 (4H, m, 4-Py), 8.71 (8H, br. s, pyrrole), 8.82 (8H, s, 2,6-Py). ^13^C NMR (CDCl_3_ δ, ppm): 172.4, 150.7, 146.1, 144.5, 138.6, 134.8, 127.6, 119.8, 118.7, 61.6, 33.3, 30.5, 25.0, 23.6. Elemental analysis: found, %: C 62.58; H 5.35; N 6.45, C_88_H_86_N_8_O_8_Br_4_ calculated, %: C 62.05; H 5.09; N 6.58. LCMS (ESI) *m*/*z*: 345.7 [M^+^-4Br]^+^; the molecular formula of C_88_H_86_N_8_O_8_ requires 345.66.

#### 3.6.6. *n*-Octanol/PBS Partition Coefficients

The distribution coefficient was measured using 1-*n*-octanol and phosphate buffered saline (PBS), which were presaturated with each other by the described method [53]. A portion of porphyrin (1–1.5 mg) was added to saturated solutions of 1-*n*-octanol (3 mL) and PBS (3 mL), sonicated and shaken for 30 min, then centrifuged to separate the layers. The absorption of each layer was measured spectrophotometrically in the region of the Soret band. Partition coefficients were calculated as the ratio between absorption in the Soret band of photosensitizers in organic and aqueous phase and the dilution factors for the organic and aqueous layers, according to the aforementioned method.

### 3.7. Statistical Analysis

Graphing, as well as statistical processing of the results, were performed using Microsoft Excel 2007 (Albuquerque, NM, USA) and GraphPadPrism v. 7.00 (San Diego, CA, USA). To assess the statistical significance, we used a two-pair *t*-test.

## 4. Conclusions

A simple and convenient approach for the synthesis of new cationic *meso*-arylporphyrins has been proposed. For solubilization of the monomeric form of porphyrins Pluronic F-127, polymer micelles were used. It has been shown that the studied cationic porphyrins, which are potential photosensitizers, exhibited a pronounced antiherpetic effect without additional light exposure. Porphyrin **3a** was found to have low toxicity and showed an antiviral effect only at high concentrations, while an increase in the length of aliphatic spacer sites between the macrocycle and cationic centers in compound **3b** increased the toxicity of porphyrin. In this case, porphyrin **3b** exhibited an antiviral effect at lower concentrations and in a wider range of concentrations. The value of IC_50_, µM/L for porphyrin **3b** was found to be more than 10 times lower than CC_50_, µM/L, which indicates the prospects of this substance for further studies. The known cationic porphyrin **TMePyP** exhibited antiviral activity against HSV-1 in a wide range of concentrations. Compounds **3a**, **3b** and **TMePyP** showed both a virucidal effect and an inhibitory effect on the reproduction of HSV-1. The use of Pluronic F-127 as a nanoscale delivery vehicle did not increase the antiherpetic activity of the porphyrins used. However, its use may have a positive effect on the photodynamic activity of porphyrins in future studies. The obtained compounds are promising for the development of utilitarian antiviral agents and, possibly, medical antiviral drugs. 

## Figures and Tables

**Table 1:** Photophysical parameters and distribution coefficient of compounds **3a,b**.

No.	λ_abs_ (nm) (ε 10^−3^ L mol^−1^ cm^−1^) ^a^					λ_em_ (nm)		Φ*_F_* ^b^	Stokes Shift	LogP ^c^
	Soret	QI	QII	QIII	QIV	Q (0,0)	Q (0,1)			
3a	415 (312)	512 (14.5)	546 (6.2)	590 (3.7)	645 (3.5)	651	721	0.14	238	−4.86
3b	418 (328)	513 (13.37)	548 (6.07)	592 (3.64)	648 (3.56)	652	723	0.13	236	−4.6

^a^, ε: extinction coefficient; ^b^, Φ*_F_*: quantum fluorescence yield is determined using TPP in dimethylformamide (DMF) (Φ*_F_* = 0.11) as a standard; ^c^, Log*Pow*: 1-*n*-octanol/water partition coefficients (pH 7) (*v*/*v*).

**Table 2:** CC_50_ and IC_50_ values of porphyrins dissolved in phosphate-buffered saline (PBS) or Pluronic F-127.

Compounds	CC_50_, µM		IC_50_, µM	
	**PBS**	**F127**	**PBS**	**F127**
**3a**	>100	>100	53.6	99.8
**3b**	43.0	27.0	3.4	3.6
**TMePyP**	>100	>100	2.3	13.3

## Data Availability

The data presented in this study are available within the article, the associated Supplementary Materials, or on request from the corresponding author.

## Supplementary Materials

The following are available at https://www.mdpi.com/1424-8247/14/3/242/s1, spectral data of synthesized compounds and data for virucidal activity is available.
