# Supplementary material for: Novel Cationic Meso-Arylporphyrins and Their Antiviral Activity against HSV-1
Source: Pharmaceuticals (Basel). 2021 Mar 8;14(3):242. doi: 10.3390/ph14030242 (PMC7999199; doi:10.3390/ph14030242)
Supplement: Supplementary file 1 [file pharmaceuticals-14-00242-s001.pdf]

Supplementary Materials

Novel Cationic meso-Arylporphyrins and Their Antiviral Activity Against HSV-1

Kseniya A. Zhdanova[1,*], Inga O. Savelyeva[1], Artem V. Ezhov[1], Andrey P. Zhdanov[2], Konstantin Yu. Zhizhin[1,2], Andrey F. Mironov[1], Natal'ya A. Bragina[1], Alla A. Babayants[3], Irina S. Frolova[3], Nadezhda I. Filippova[3], Nadezhda N. Scliankina[3] and Olga N. Scheglovitova[3]

[1] MIREA – Russian Technological University, Vernadsky prospect 86, Moscow, 119571, Russian Federation; e-mail@e-mail.com
[2] Kurnakov Institute of General and Inorganic Chemistry of the Russian Academy of Sciences, Leninskii pr. 31, Moscow, 117907 Russia
[3] Gamaleya Research Center of Epidemiology and Microbiology, Gamaleya str., 18, Moscow, 123098, Russian Federation
* Correspondence: zhdanova_k@mirea.ru; Tel.: +79261126692 K.A.Z.

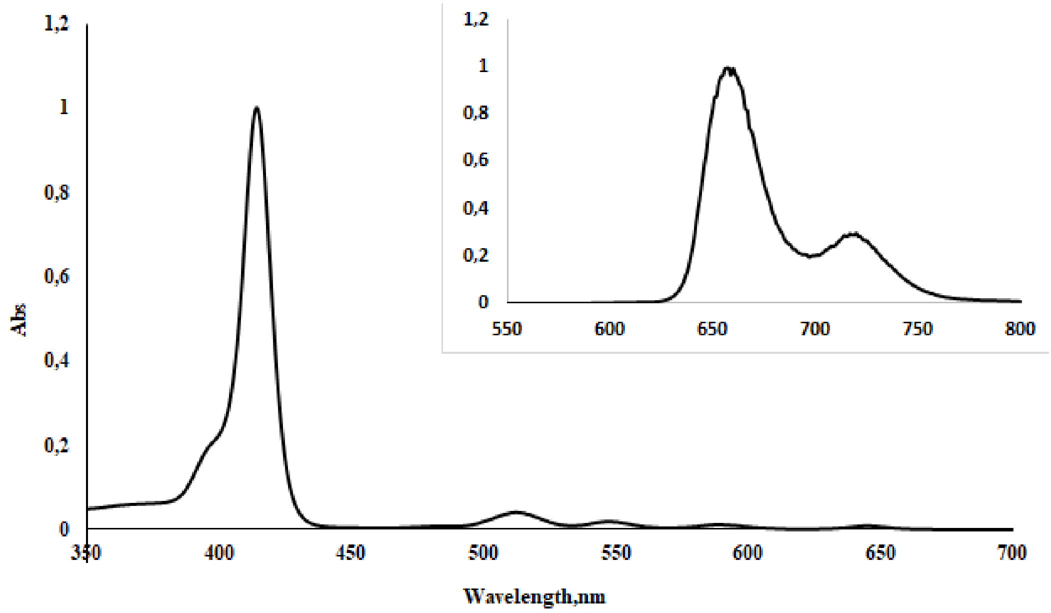

Figure s1. Electronic absorption spectrum of **3a** in DMSO. Fluorescence spectrum is shown in the insert.

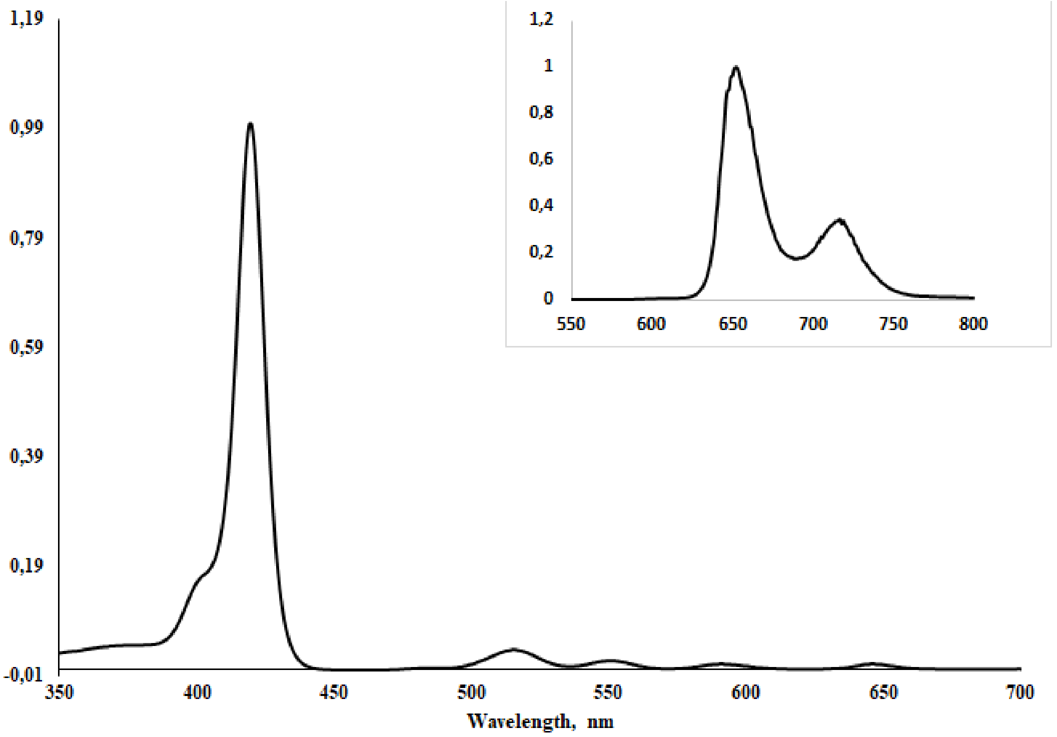

Figure s2. Electronic absorption spectrum of **3b** in DMSO. Fluorescence spectrum is shown in the insert.

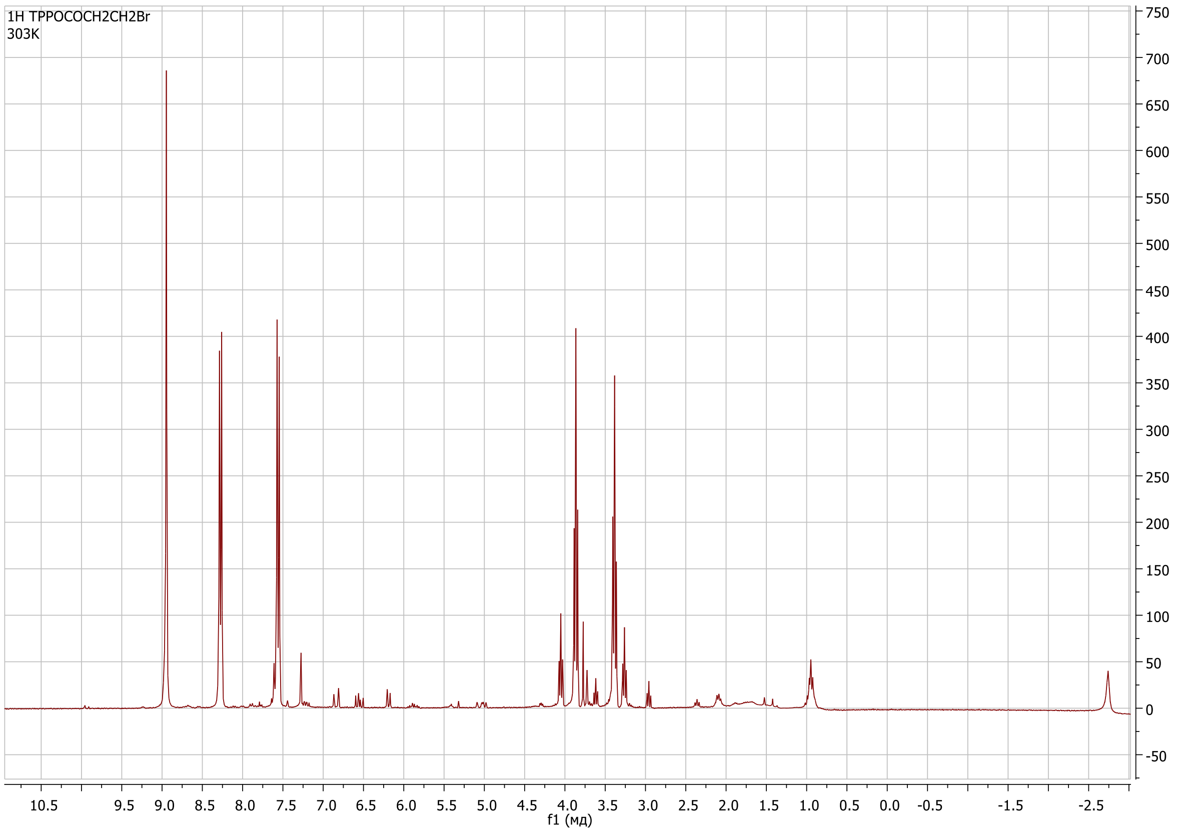

Figure s3. ^{1}H NMR spectrum for **2a**

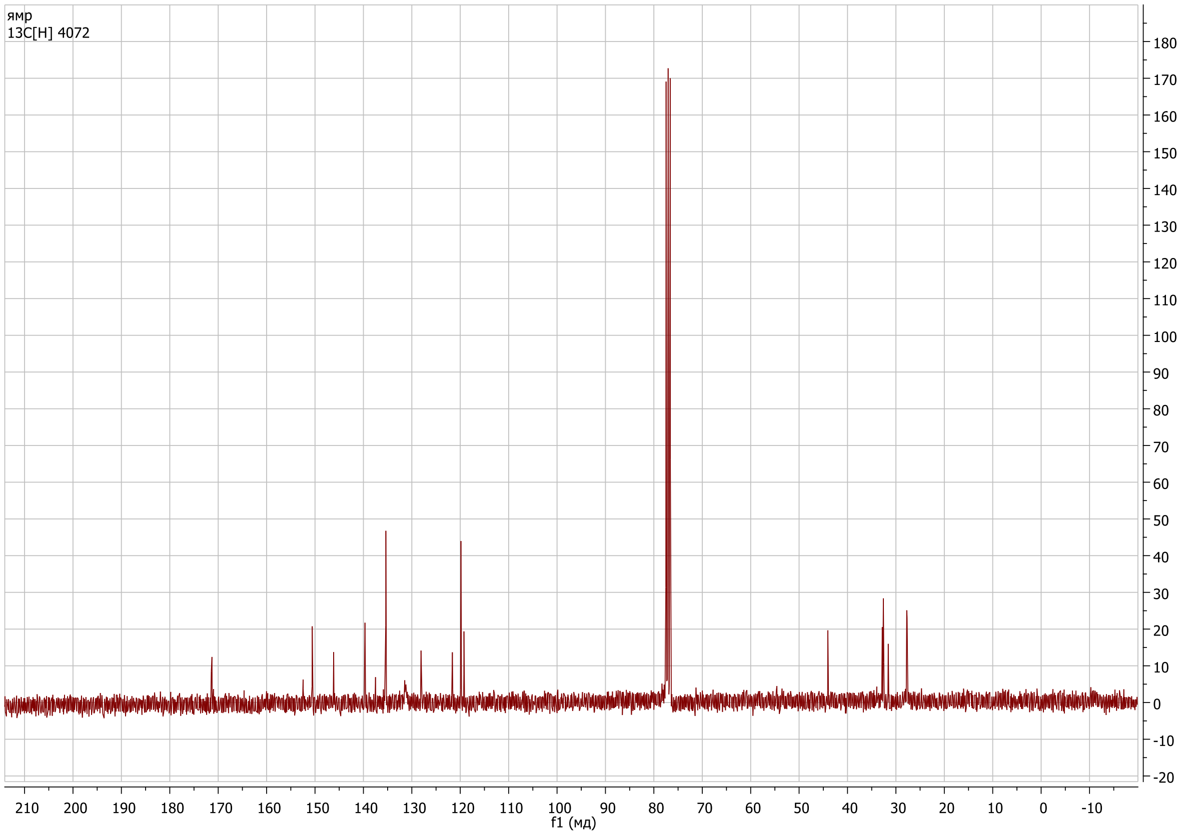

Figure s4. ^{13}C NMR spectrum for **2a**

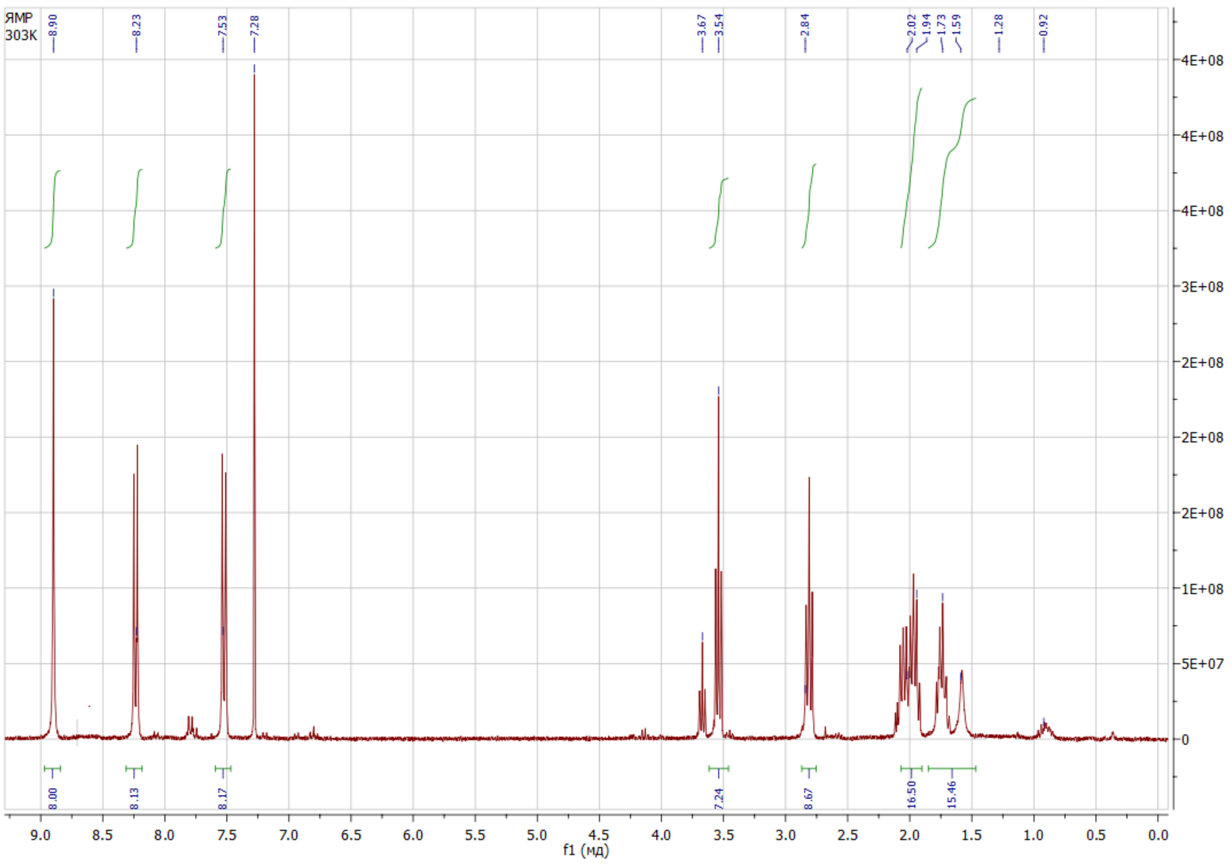

Figure s5.¹H NMR spectrum for **2b**

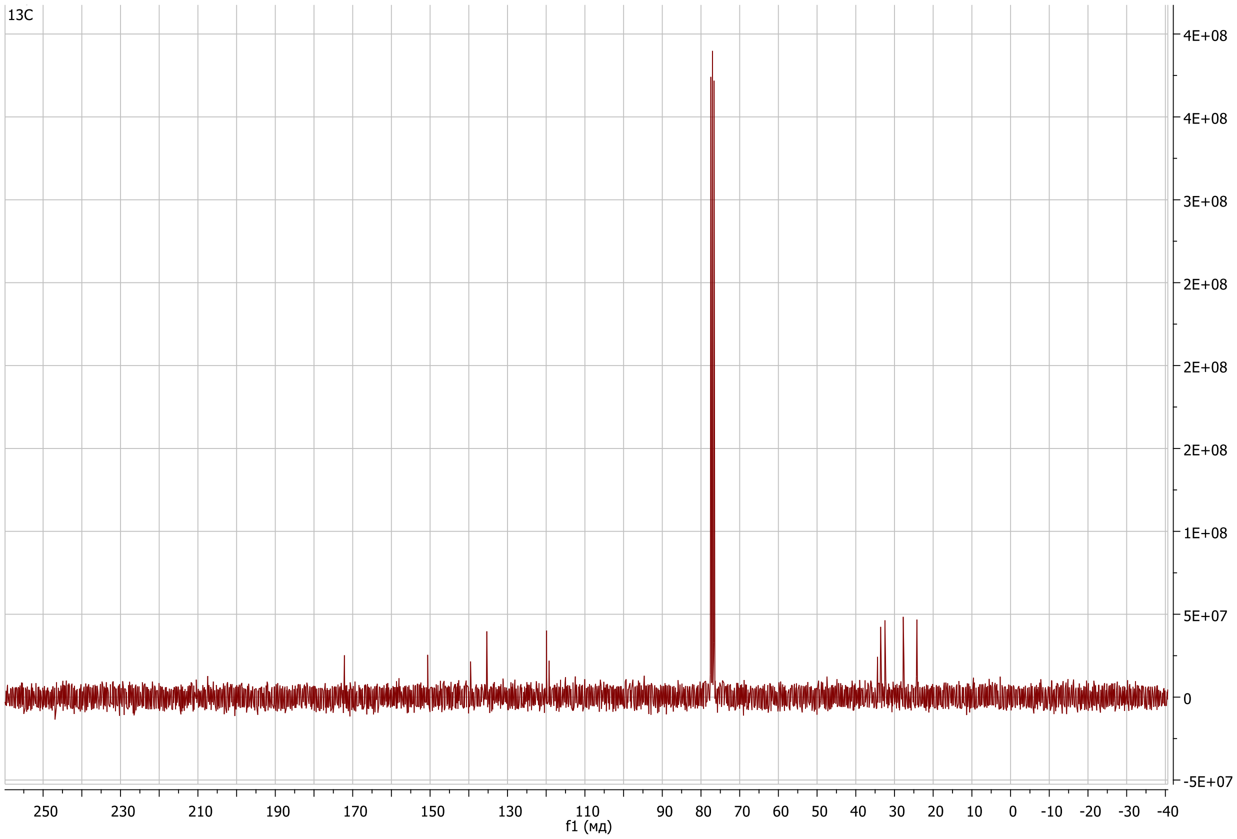

Figure s6.¹³C NMR spectrum for **2b**

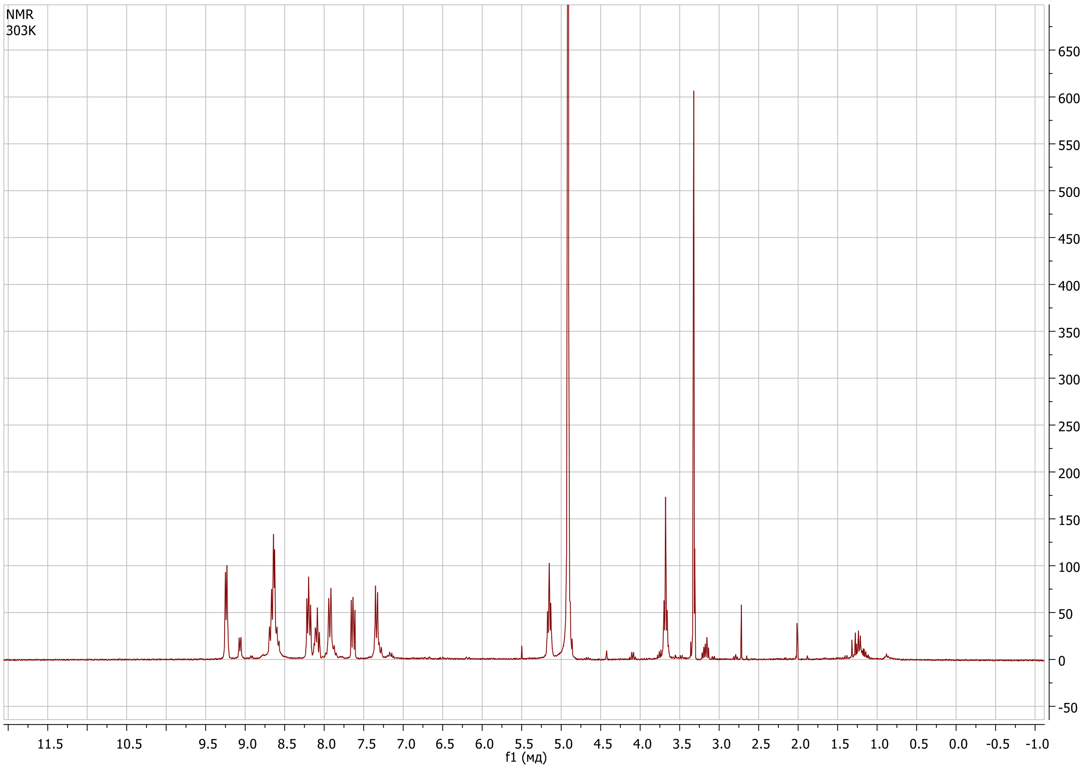

Figure s7.^{1}H NMR spectrum for **3a**

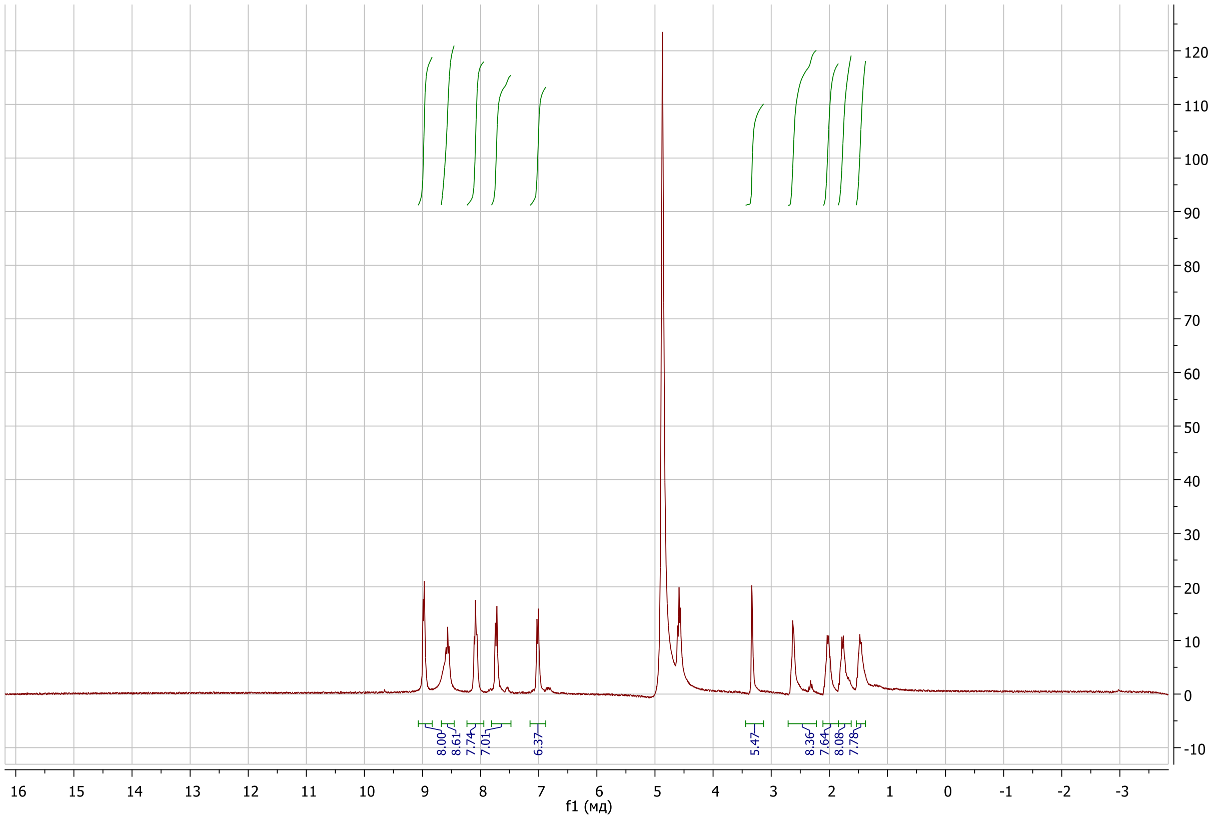

Figure s8.^{1}H NMR spectrum for **3b**

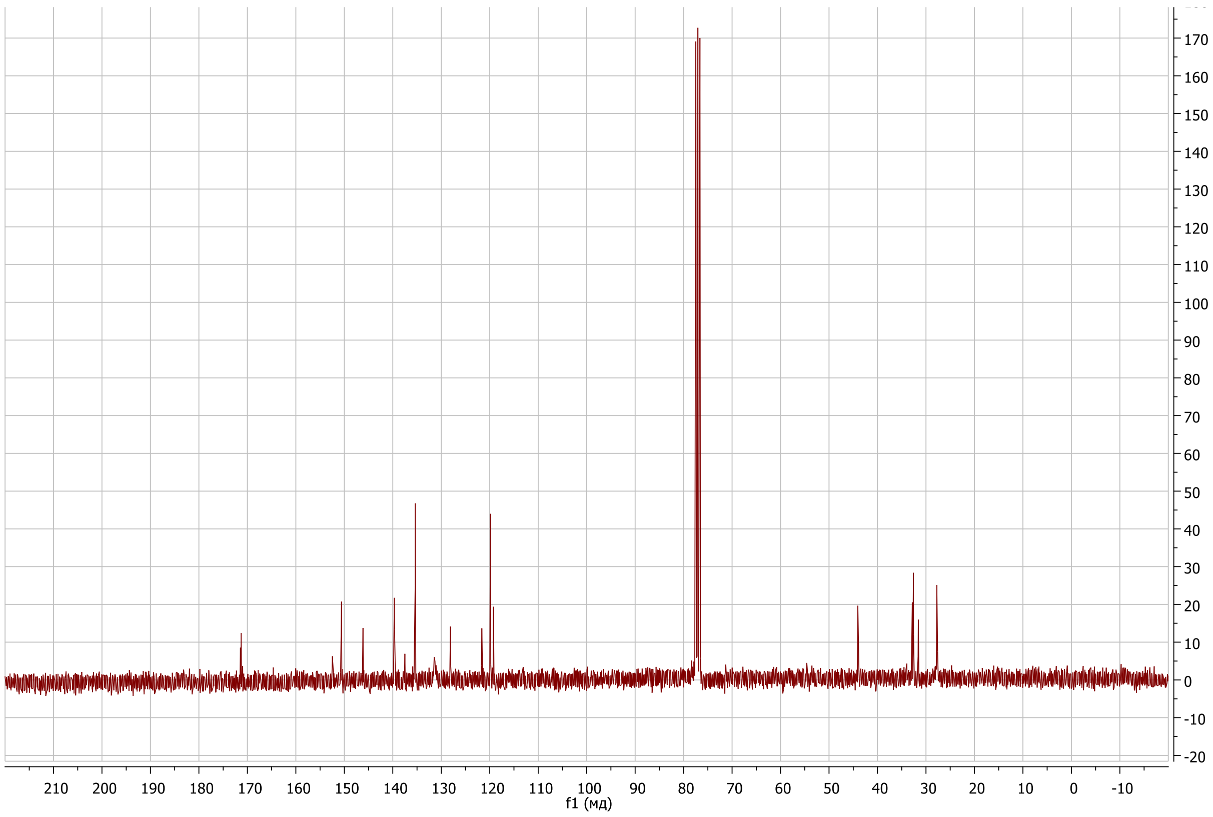

Figure s9. ^{13}C NMR spectrum for **3a**

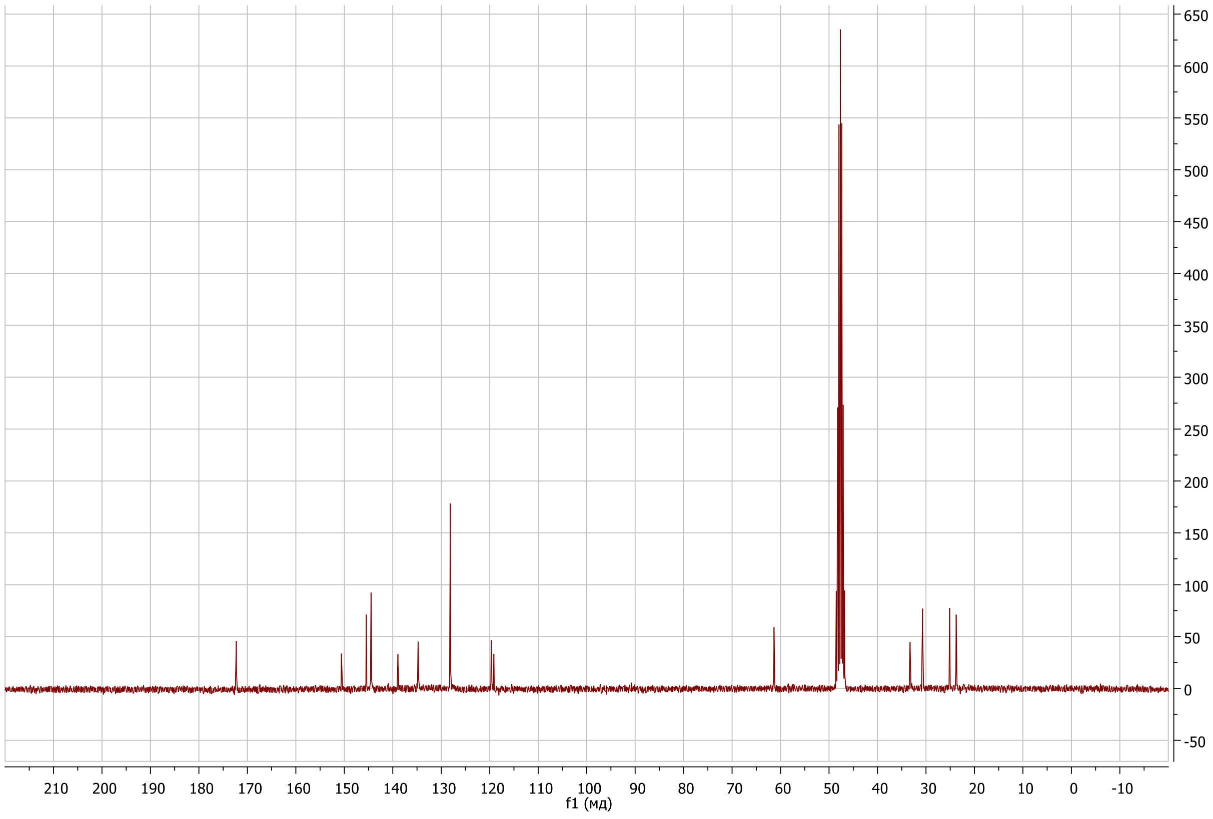

Figure s10. ^{13}C NMR spectrum for **3b**

Fragmentor Voltage 135 **Collision Energy** 0 **Ionization Mode** ESI

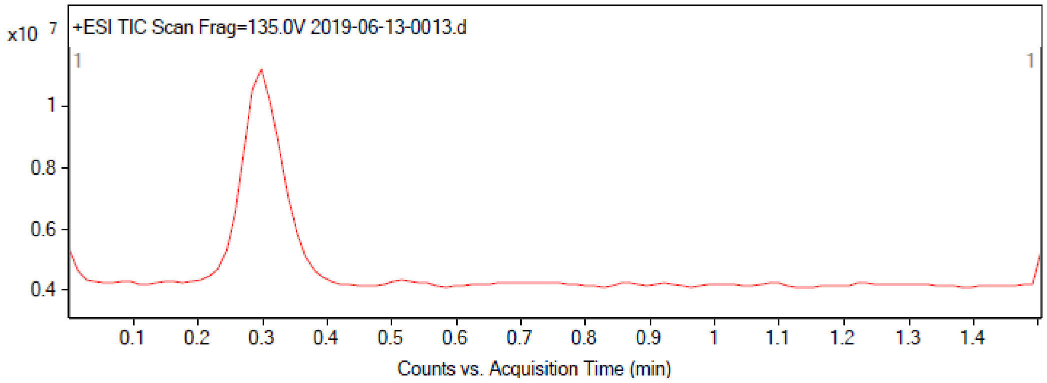

Qualitative Analysis Report

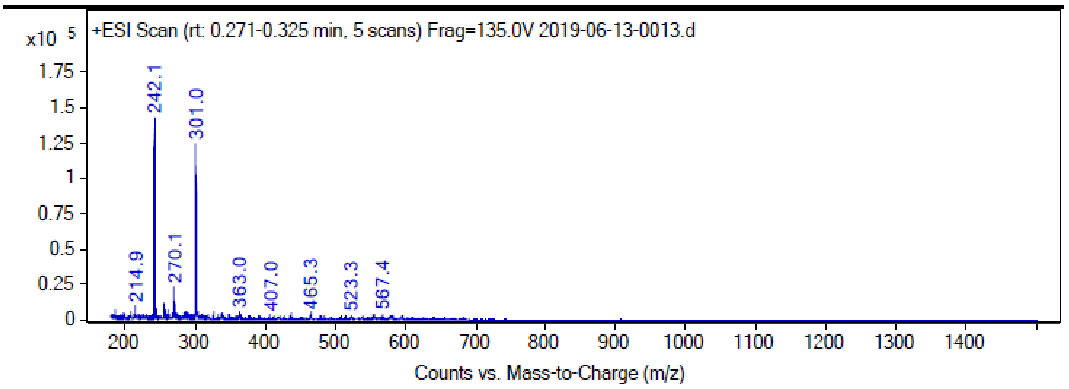

Figure s11. ESI-MS spectrum for **3a.**

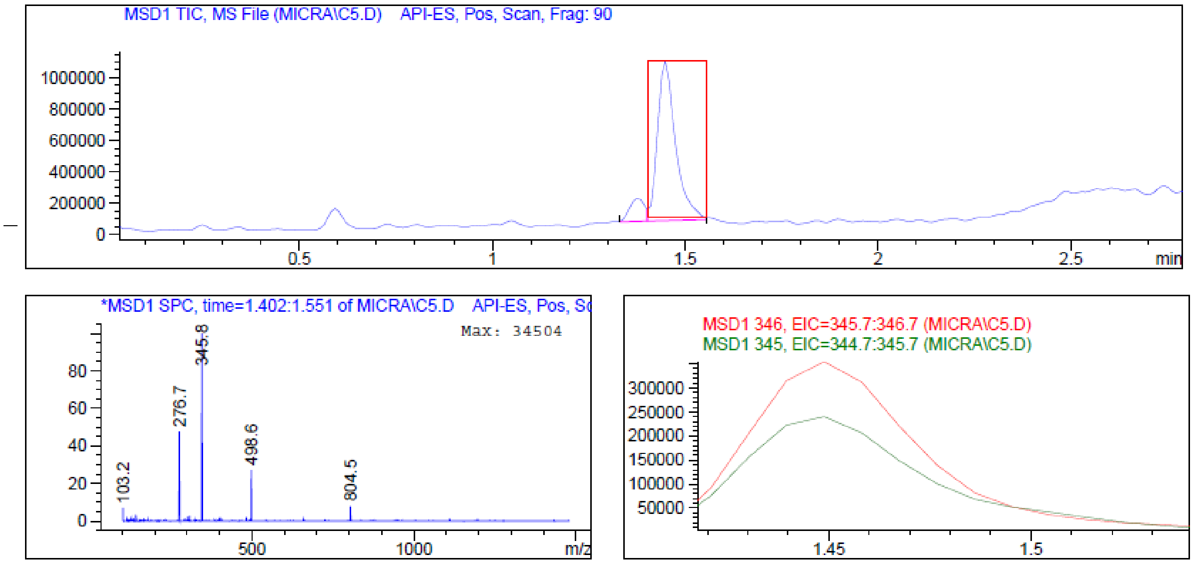


```
Peak #2 at  1.448 min ( 1.403 to  1.554 min)
-> The analysis found only one component, indicating a pure peak. <-

Component 1:  Peak at Scan 152.9. Top ions are  346 345 277
```

Figure s12.ESI-MS spectrum for **3b.**

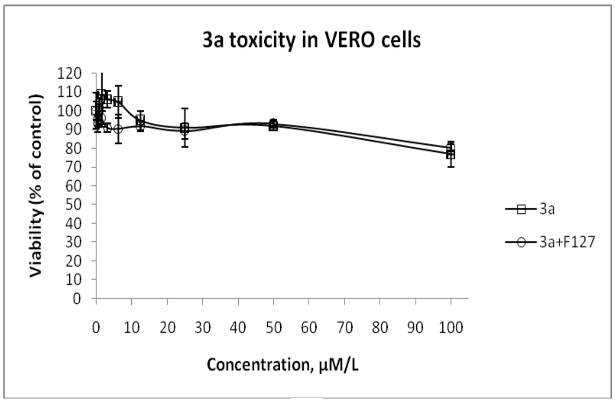

(a)

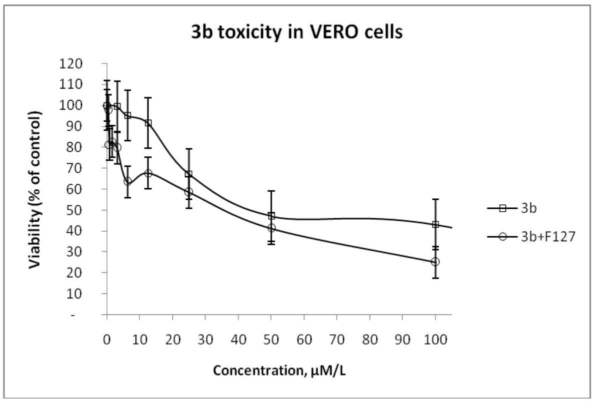

(b)

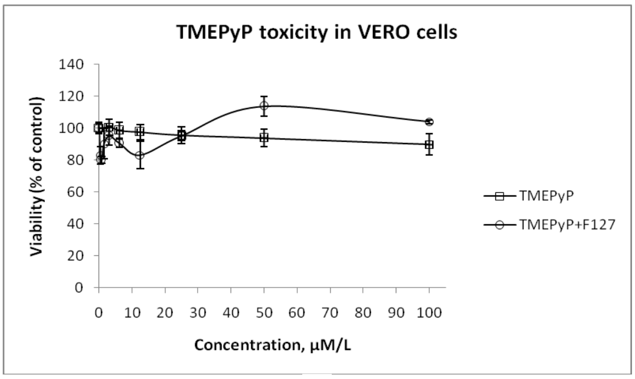

(c)

Figure s13. Toxicity of compounds **3a** (a), **3b** (b), **TMePyP** (c), dissolved in PBS and F127, in Vero cell culture (MTT test). The results are presented as the living cells percentage relative to the living cells in control, which was taken as 100%. Each point represents the mean ± standard deviation of two experiments were performed with four replicates each.

Table s1. Vero cell viability after exposure to **3a**, **3b**, **TMEPyP** compounds dissolved in PBS and Pluronic F-127. Different concentrations of compounds were applied to the cell monolayer and after incubation for 3 days, the number of living cells was calculated in the MTT test. Each point represents the mean ± standard deviation of three experiments with forth replicates each. n.d. –not done.

**Compound**	**Concentration, μM/L**	**Viability % ±s.d.**	
		**-F127**	**+F127**
3a	0.39	94.6±2.9	93.0±4.6
	0.78	102.4±4.2	99.2±4.2
	1.56	108.3±15.4	95.6±4.2
	3.13	106.0±4.5	90.8±2.3
	6.25	104.6±8.7	90.3±7.8
	12.50	94.7±5.1	91.7±2.8
	25	90.9±10.0	89.1±4.1
	50	91.9±2.2	92.9±2.0
	100	76.8±6.9	80.4±1.8
3b	0.39	n.d.	97.6±6.9
	0.78	n.d.	81.3±10.8
	1.56	n.d.	82.7±19.5
	3.13	99.5±2.9	79.6±9.6
	6.25	95.2±3.6	63.4±8.1
	12.50	91.6±5.0	67.7±11.5
	25	67.2±2.1	58.4±4.4
	50	47.1±2.5	41.3±2.7
	100	43.1±2.4	25.0±1.9
	200	8.0±0.4	
TMEPyP	0.39	n.d.	82.9±5.4
	0.78	n.d.	79.9±2.3
	1.56	n.d.	90.2±9.7
	3.13	100.6±5.0	95.2±6.0
	6.25	98.5±5.0	90.4±2.7
	12.50	97.4±4.6	83.0±8.6
	25	95.3±5.3	95.2±3.2
	50	93.7±5.5	113.6±6.2
	100	89.6±6.8	103.8±1.2

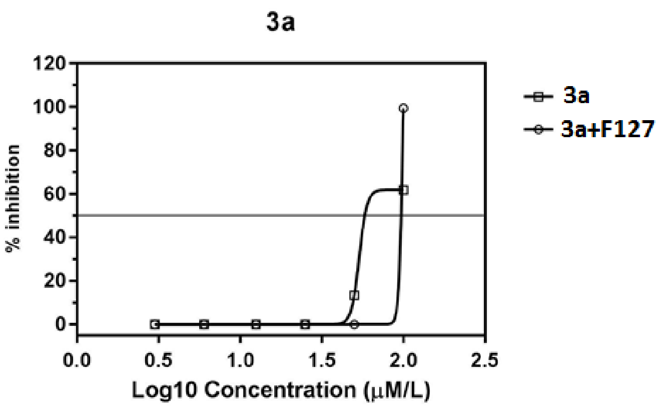

(a)

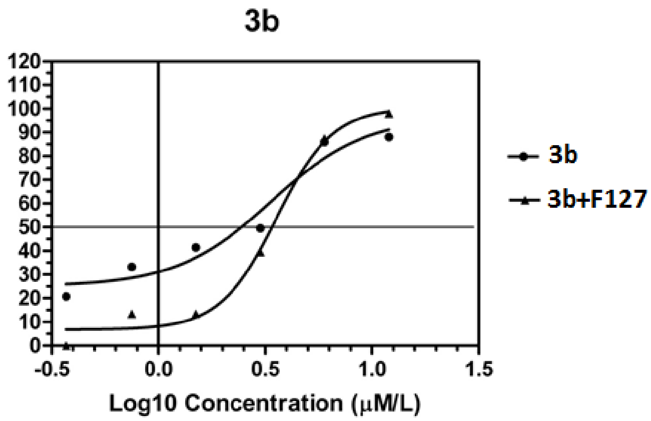

(b)

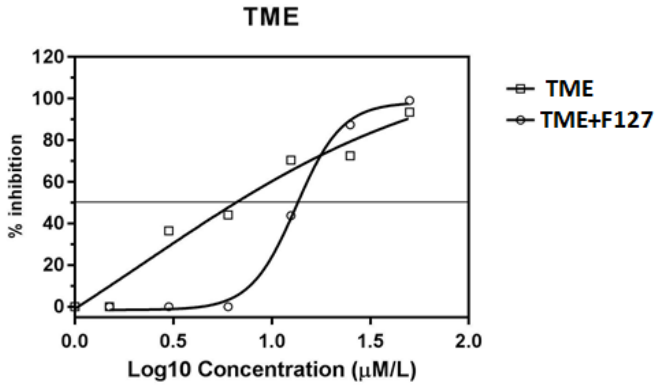

(c)

Fig. 15S. Virucidal activity of **3a** (A), **3b** (B), **TMePyP** (C) compounds dissolved in PBS and Pluronic F127 regarding to HSV-1. HSV-1 at tenfold dilutions DMEM-1%FCS was incubated with different concentration of the compounds ranging from 3 μM/L to 100 μM/L in DMEM-1% FCS or without of compound , each sample in volume 1mL, for 2 hours at 36.8°C in 5% CO_2 atmosphere. After treatment the samples were applied to the Vero DMEM-5%FCS in 96 wells plates) monolayers in 96 wells plates (100 μL/well). and after 4 days of cultivation at 36.8 °C in 5% CO_2 atmosphere titers were calculated based on the development of CPE and the inhibitory activity of each compounds was calculated in relation to the control (HSV-1 without compounds). Data are reported as means of three independent assays, each run in triplicate. The results were processed using the GraphPad Prism 5 program.
